# Exploring the HIV Disclosure Patterns to Sexual Partners and Associated Factors Among HIV-Positive Adults in Sheger City, Ethiopia: A Multicenter Study

**DOI:** 10.1155/arat/4117734

**Published:** 2025-04-17

**Authors:** Firaol Regea Gelassa, Mesfin Hailu Shene, Takele Tiki Kejela, Tesfu Zewdu Gemmeda, Elias Andasha Fana, Lammi Atomsa, Tsegae Benti Muse

**Affiliations:** ^1^Department of Nursing, School of Health Sciences, Ambo University, Waliso Campus, Waliso, Ethiopia; ^2^Department of Public Health, Oromia Health Office, Addis Ababa, Ethiopia; ^3^Department of Psychiatry, College of Medicine and Health Sciences, Ambo University, Ambo, Ethiopia; ^4^Department of Nursing, College of Health Sciences, Salale University, Fiche, Ethiopia; ^5^Department of Midwifery, School of Health Sciences, Ambo University, Waliso Campus, Waliso, Ethiopia; ^6^Department of Nursing, School of Nursing and Midwifery, College of Health Sciences, Wollega University, Nekemte, Wollega, Ethiopia; ^7^Department of Public Health, College of Medicine and Health Sciences, Ambo University, Ambo, Ethiopia

**Keywords:** adults, disclosure, HIV status, sexual partner, Sheger city

## Abstract

**Background:** Disclosing HIV serostatus to a partner is essential for HIV prevention and care. It encourages safer sexual practices, lowers the risk of transmission, and helps individual's access to treatment and support. However, the choice to share one's HIV status can be affected by a range of personal and societal influences. Ethiopia has a diverse population where traditional norms and health challenges intersect which might negatively influence HIV disclosure. Therefore, this study aims to explore HIV disclosure patterns to sexual partners and associated factors among HIV-positive adults in Sheger City, Ethiopia.

**Methods:** An institution-based cross-sectional study was conducted among 393 people living with HIV attending the ART clinic in Sheger City from August 1 to September 30, 2023. Study participants were selected using a systematic sampling technique. Data were collected through a pretested, interviewer-administered structured questionnaire. Multicollinearity was assessed using the variance inflation factor (VIF). To evaluate the goodness of fit of the logistic regression model, we calculated the pseudo-*R*^2^ values and the area under the receiver-operating characteristic (ROC) curve. Binary and multivariable logistic regression analyses were performed to identify factors independently associated with HIV disclosure status. Adjusted odds ratios (AOR) with 95% confidence intervals (CI) were calculated, and statistical significance was determined at a *p*-value of < 0.05.

**Results:** The overall prevalence of HIV serostatus disclosure to sexual partners was 67.9% (95% CI: 63.5%, 72.5%). Factors significantly associated with HIV disclosure included pretest counseling (AOR = 7.86; 95% CI: 3.61, 17.08), marital status (AOR = 9.32; 95% CI: 2.62, 33.19), presence of initiating factors (AOR = 7.18; 95% CI: 3.41, 15.01), type of testing (AOR = 6.44; 95% CI: 2.43, 17.07), perception of HIV-related stigma (AOR = 0.21; 95% CI: 0.09, 0.47), and having clinical symptoms at the time of HIV testing (AOR = 22.12; 95% CI: 8.74, 56.20).

**Conclusion:** This study found that 67.9% of people living with HIV disclosed their serostatus to their sexual partners. Pretest counseling, being married, the presence of initiating factors, self-initiated testing, and experiencing clinical symptoms during testing were found to be positively associated with HIV status disclosure. In contrast to this, the perception of HIV-related stigma was associated with lower rate of disclosure. Thus, enhancing pretest counseling, launching community-based initiatives and offering extra support for symptomatic individuals are essential strategies to increase disclosure rates.

## 1. Background

Acquired immune deficiency syndrome, or AIDS, is an important concern for public health worldwide as well as in Ethiopia [1]. Recent data indicate that Ethiopia bears a substantial HIV/AIDS burden, with an estimated prevalence rate of approximately 0.8% among adults aged 15–49. The epidemic is more pronounced in urban areas. Despite ongoing initiatives, significant barriers persist in terms of access to testing, treatment, and reducing stigma [2].

Management of HIV involves not only the prevention of new infections but also the proper care of those infected to prevent further transmission [3]. In HIV prevention strategy, the issue of human immunodeficiency virus (HIV) status disclosure is very important because of its potential effects on prevention and its relation to confidentiality as a human right. Disclosure is the process of revealing HIV-positive status to sexual partner(s), family members, friends, or others in their social circle but not to healthcare providers [4].

Effective HIV therapy and prevention necessitate the disclosure of HIV to sexual partners. It enhances access to therapy, promotes safer sexual practices, and uplifts the psychological health of individuals impacted [5, 6]. A common concern within HIV prevention is that HIV-positive individuals do not disclose their HIV status to their partners, who are thus at increased risk of HIV infection [6]. While disclosure can improve treatment adherence and enhance social support, it is still a complicated topic, influenced by several social, cultural, and psychological factors, despite its significance [7]. Studies conducted on different continents of the world revealed that several factors, including social, cultural, and psychological factors, as well as fear and stigma, are those factors that significantly affect the HIV serostatus status [7–11].

The World Health Organization has also recommended that all HIV-positive patients should immediately disclose their HIV-positive status to their prospective sexual partners to motivate partners for voluntary counseling and testing (VCT), reduce risk behaviors, and increase the acquisition of support and adherence to ART [12, 13].

Ethiopian cultural norms, stigma, and healthcare procedures may influence the dynamics of disclosure, especially in urban environments like Sheger City. It is essential to comprehend the trends and factors that influence HIV disclosure in this setting to create focused interventions and support networks. There is no legal concern in Ethiopia about HIV disclosure status. In some other developed countries, however, disclosure of HIV-positive status is regarded as a social and legal responsibility for people living with HIV [14].

Even though people living with HIV/AIDS (PLWHA) are given knowledge about safer sexual behavior during the ART clinic sessions, there is growing evidence suggesting that people on ART are increasingly becoming sexually active, and many of them are involved in sexual activity with partners who are HIV-negative [15]. Some of these people practice unsafe sexual behavior patterns such as not using condoms and having multiple sexual partners without disclosure of their serostatus. These put them at risk of contracting re-infections with another strain of the virus and lead to problems with drug resistance [15].

Despite the significant importance of the HIV serostatus disclosure to sexual partners to improve social support and treatment adherence, to the level of our search engine, there is a limited study that explores the patterns of HIV serostatus disclosure to their sexual partner in Ethiopia in general and in study areas particularly. Therefore, the purpose of this study is to investigate the patterns and variables influencing HIV disclosure to their sexual partner in Sheger City among people who are HIV-positive. Considering that developing focused initiatives to raise disclosure rates and strengthen general HIV care and prevention strategy requires an understanding of these processes.

## 2. Methods and Materials

### 2.1. Study Setting and Populations

A facility-based cross-sectional study was conducted in public health facilities in Sheger City, Ethiopia, from August 1, 2023, to September 30, 2023. Sheger City, officially established in 2022, is part of Oromia Regional State and surrounds Addis Ababa. Sheger City is one of the cities in Oromia that comprise six various districts. It is located surrounding Addis Ababa, the capital city of Ethiopia. The total population of Sheger City is estimated to be 794,489. It is bordered on the south by the city of Addis Ababa, on the west by the Mulo and East Shewa Zone, on the north by the North Shewa Zone, and on the east by the Berek district [16].

The study populations were all systematically selected adult people living with HIV who were on antiretroviral therapy (ART) at public health facilities in Sheger City. We included all adults aged 18 years or older who had a sexual partner at the time of HIV diagnosis and were on ART follow-up care for at least one-month duration during the data collection period and excluded HIV-positive individual who started ART during the study period. Individuals who recently started ART were excluded to ensure participants had sufficient time to engage in disclosure processes. Considering the inclusion of recent initiates could compromise the reliability of disclosure data due to the limited time for disclosure dynamics to develop.

### 2.2. Sample Size and Sampling Procedures

The sample size was calculated using the single population proportion formula, based on a 52.6% disclosure rate from previous studies in Debre Markos, Ethiopia [4], with a 5% margin of error and a 5% level of significance at a 95% confidence interval. Including a 10% adjustment for nonresponse rate, the final sample consisted of 422 adults living with HIV in the study area.

Before the main study, two [2] districts and one [1] administrative town were selected using a lottery method (i.e., Mulo district, Sululta district, and Sendafa town, respectively). The total sample size was allocated to each health facility by proportional allocation to size based on the number of eligible individuals they had. Then, eligible individuals in the selected health facilities were enumerated from the medical registration logbook of patients at ART follow-up to ensure a comprehensive and well-defined sampling frame. First, we counted the number of HIV follow-ups in one-quarter and then calculated the daily flow of HIV-positive participants based on the previous quarter's follow-up data. Using this flow, we projected the 2-month flow and divided the total 2-month flow by the desired sample size. This method helped mitigate potential bias. Finally, four hundred twenty-two (422) participants were enrolled in the study using a systematic sampling technique.

### 2.3. Data Collection Tool and Procedures

A structured questionnaire was developed based on previously validated tools and relevant literature [17, 18] (See Supporting File 1). To ensure the reliability of the instrument, internal consistency was assessed, yielding a Cronbach's alpha value of 0.83, indicating a high level of interrelatedness and cohesiveness among the items. The questionnaire was initially designed in English, and then translated into Amharic and Afan Oromo, followed by a back-translation into English to maintain accuracy and consistency.

A pretest of the structured questionnaire was conducted before the actual data collection on 5% individuals in the adjacent health center. Accordingly, appropriate amendments were made to the questionnaire after the pretest. All the questionnaires were checked daily to ensure that they were appropriately filled or not. The questionnaire collected data on sociodemographic variables including sex, age, educational status, religion, marital status, occupation, and personal and institutional characteristics, using standardized tools [17–20] designed to assess status disclosure after establishing reliability.

Data collection was conducted by six [6] BSc nurses who were employed at ART clinics. They administered the pretested structured questionnaire to individuals attending ART services at selected ART sites. Two BSc public health officers supervised the data collection. Both data collectors and supervisors received training. Prior to data collection, participants were given an orientation on the study's purpose. Voluntary informed written consent was obtained from all participants. To mitigate the social desirability bias and recall bias, participants were reassured of the confidentiality of their responses to minimize socially desirable answers.

Data collectors were trained to create a neutral and nonjudgmental interview environment and only participants who had been on ART for at least 1 month to ensure recall accuracy. Additionally, questions were structured chronologically to assist participants in recalling events systematically. In addition to this, the data collection processes were closely followed by the supervisors and the principal investigator daily. The supervisors and principal investigator performed immediate supervision daily to ensure each completed questionnaire was really completed. Finally, the data were edited for possible errors, double-entered into EpiData Version 3.1 to control for errors that occurred during data entry, and cleaned for missing values and outliers in SPSS Version 25.

### 2.4. Measurements

*Disclosure to partner*: The act of disclosing one's HIV result to a sexual partner [17].

*No disclosure*: When the HIV-positive patient tells nothing about his or her illness [17].

*HIV-positive individual on ART*: People who are living with HIV/AIDS and who had at least one visit to the selected ARV treatment care for receiving ARV treatment [18].

### 2.5. Data Analysis

The collected data were edited, coded, and entered into EpiData Version 3.1 and then exported to SPSS 25 for analysis. Missing data were assessed before analysis. Since the proportion of missing data were less than 5%, we used complete case analysis, assuming data were missing at random. No imputation techniques were applied as messiness was minimal. Frequencies, proportions, and summary statistics were computed to describe the study population in tables, graphs, and charts. Bivariable and multivariable logistic regression were computed to determine the presence and degree of association between the independent and dependent variables. In the bivariable analysis, variables with a *p*-value < 0.25 were included in the multivariable logistic regression model. This threshold is commonly used to avoid excluding potentially important predictors that may become significant when adjusted for other covariates, and a *p* value < 0.05 and 95% CI were used to judge statistical significance. Model diagnostics were conducted to assess the adequacy of the logistic regression model. The Hosmer–Lemeshow test was used to evaluate goodness of fit, and the *p*-value was 0.64 indicating a good model fit. Additionally, the goodness of fit for the logistic regression model was assessed using Nagelkerke's (*R*^2^ = 0.52), indicating that approximately 52% of the variance in the outcome variable was explained by the independent variables. Multicollinearity was assessed using the variance inflation factor (VIF), with values reported for all independent variables. The VIF values ranged from 2.77 to 6.90, all below the commonly accepted threshold of 10, indicating no significant collinearity among the predictors. The details of the VIF value for all independent variables have been included in the table (see Table 1).

## 3. Results

### 3.1. Sociodemographic Characteristics of the Participants

A total of 393 participants were enrolled in the study, yielding a response rate of 93.13%. More than half (55.2%) were female. Nearly half (49.4%) were aged 25–34 years, with a mean age of 33.2 years (SD ± 8.5). The majority (62.8%) were married. Additionally, 44.3% had no formal education, and the largest occupational group comprised housewives (31%, *n* = 122) (see Table 2).

### 3.2. Psychosocial and Sexual Characteristics of the Participants

HIV testing and disclosure patterns varied among the study participants. About 31.3% of participants undergo HIV testing and received their results during antenatal care (ANC) follow-up, and 107 participants (27.2%) were tested through provider-initiated voluntary counseling.

Regarding sexual partnerships, the majority (87.8%, *n* = 345) reported having only one sexual partner. In terms of HIV testing accompaniment, 298 participants (75.8%) attended testing alone, whereas 86 (21.9%) were accompanied by their partner, which may reflect variations in partner support and involvement in HIV-related healthcare decisions. This study shows that the relationship dynamics also played a role in HIV disclosure. Among the participants, 280 (71.2%) described their relationship with their sexual partner as smooth, while 113 (28.8%) reported challenges in their relationship.

HIV-related stigma was a notable concern, with 140 participants (35.6%) perceiving stigma associated with their status, which could impact their willingness to disclose and seek support. Concerning their partner's HIV status, 217 participants (55.2%) reported having an HIV-positive partner, while 68 (17.3%) had an HIV-negative partner. Notably, 108 participants (27.5%) were unaware of their partner's HIV status, emphasizing gaps in partner communication and testing practices (see Table 3).

### 3.3. Behavior-related HIV Status Disclosure and Partner Reaction Characteristics

In this study, more than half of the participants (51.7%) reported consuming alcohol up until the time of data collection, while less than a quarter used substances like Khat (13.3%) and cigarettes (8.8%). Among those who had disclosed their HIV status, 176 (65.9%) received support from their family members, while 91 (34.1%) experienced discrimination from their families. Additionally, 76 (28.5%) were bothered by others after disclosing their status.

Of the total participants, 303 (77.1%) reported using condoms before disclosing their HIV status, citing various reasons: 278 (70.7%) to prevent transmitting HIV to their partner, 57 (14.5%) to prevent pregnancy or avoid having more children, and 97 (24.7%) did not use condoms at all. Furthermore, 13 (3.3%) had engaged in sexual intercourse before disclosing their HIV status to their partner (see Table 4).

### 3.4. Magnitude of HIV Status Disclosure Among Adults

In this study, the overall HIV status disclosure rate among participants was 67.9% (95% CI: 63.5%, 72.5%). However, early disclosure before receiving test results was notably low, with only 38 participants (9.7%) discussing their status with their partner before testing.

**Figure 1:**
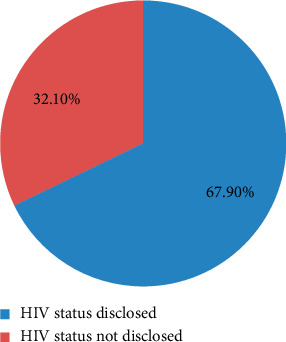
The magnitudes of HIV-positive individual disclosed their HIV status to their sexual partner among HIV-positive adults in Sheger city, Ethiopia, 2023.

Following diagnosis, 267 participants (67.9%) disclosed their HIV-positive status to their sexual partners. Beyond sexual partners, 219 participants (55.7%) shared their status with at least one family member, relative, or close friend, while 197 (50.1%) disclosed their status to individuals living with HIV/AIDS. Despite the relatively moderate disclosure rate, several barriers prevented some individuals from informing their partners. The most frequently reported reason was fear of loss of confidentiality, reported by 110 participants (41.2%). Additionally, 72 participants (27.1%) refrained from disclosure due to fear of stigma, while 40 individuals (15%) expressed concerns about potential divorce or intimate partner violence following disclosure (see Figure 1).

### 3.5. Factors Associated With HIV Disclosure Status to Sexual Partners

In a multivariable logistic regression analysis, several factors were significantly associated with HIV status disclosure to sexual partners among adults living with HIV in Sheger City. These factors included receiving precounseling related to disclosure, marital status, initiating factors for HIV testing such as social media exposure (TV, radio), the type of VCT, and the presence of clinical symptoms during VCT, and perceptions of HIV-related stigma. All of these factors were statistically associated with HIV status disclosure, with *p*-values less than 0.05.

This study found that participants who received pretest counseling the test were more likely to disclose their HIV status to their partner compared to those who did not receive pretest counseling (AOR = 7.86; 95% CI: 3.61, 17.08). Similarly, married individuals were 9.32 times more likely to disclose their status than unmarried participants (AOR = 9.32; 95% CI: 2.62, 33.19). Additionally, those exposed to initiating factors such as social media (TV, radio) and healthcare providers were 7.18 times more likely to disclose their status (AOR = 7.18; 95% CI: 3.41, 15.01) compared to those without such exposure. These findings highlight the critical role of counseling, marital support, and media influence in promoting HIV disclosure.

This study found that adults who underwent HIV testing through peer initiation were 6.44 times more likely to disclose their HIV-positive status to a partner (AOR = 6.44; 95% CI: 2.43, 17.07) compared to those who tested during routine visits. Conversely, individuals who perceived HIV-related stigma were 79% less likely to disclose their status (AOR = 0.21; 95% CI: 0.09, 0.47) compared to those who did not perceive stigma. Additionally, participants who experienced clinical symptoms at the time of testing were 22.12 times more likely to disclose their HIV status (AOR = 22.12; 95% CI: 8.74, 56.2) than those without symptoms (see Table 5).

## 4. Discussion

The objective of this study was to assess HIV disclosure status to sexual partners and the factors associated with it among HIV-positive adults on ART in health facilities providing ART services in the Sheger city. The study found that about 67.9% of HIV-positive adults disclosed their serostatus to their partners. Factors associated with HIV status disclosure in this study included receiving pretest counseling, marital status, initiating factors such as exposure to social media (TV, radio) and healthcare providers during HIV testing, the type of VCT, the presence of clinical symptoms during VCT, and perceptions of HIV-related stigma.

The magnitude of HIV status disclosure in this study is comparable to findings from studies conducted in Uganda, Amhara Regional State, Ethiopia, and Holeta, Ethiopia [4, 19, 20]. However, it is higher than results from studies in Mekelle, Ethiopia (57.4%), Axum health facilities in northern Ethiopia, and Bale Zone hospitals (52.6% and 41.8%, respectively) [13, 21, 22]. On the other hand, the magnitude of HIV status disclosure in this study is lower than findings from studies conducted in Ghana (88.7%), Debre Markos town, Ethiopia (92.6%), Jimma University specialized Hospital, Ethiopia (90.8%), and Ambo Hospital, Ethiopia (86.2%) [5, 9, 23, 24].

These variations in disclosure rates could be attributed to several contextual factors, including differences in healthcare accessibility, cultural norms, and the availability of ART services. Area with higher disclosure rates, such as Debre Markos and Jimma, may benefit from well-integrated ART services, stronger healthcare infrastructure, and higher patient–provider interactions that encourage open discussions about disclosure.

For instance, a study conducted at Debre Markos Referral Hospital highlighted that effective ART adherence and strong patient–provider interactions significantly enhance disclosure rates. Similarly, in Jimma, the presence of comprehensive ART clinics staffed with trained healthcare professionals facilitates open discussions about HIV status, thereby promoting higher disclosure [25].

In contrast, lower disclosure rates in Axum and Bale may be linked to limited healthcare access and fewer trained healthcare professionals. Studies have indicated that in these areas, factors such as perceived stigma, fear of discrimination, and inadequate counseling services contribute to lower rates of HIV status disclosure [21, 22].

On the other hand, cultural and social norms also play a significant role in disclosure behaviors. In certain communities, stigma and fear of discrimination may discourage individuals from disclosing their HIV status. Rural areas with strong traditional beliefs may have higher levels of stigma, making disclosure less likely due to the fear of social exclusion as seen in the Bale. In contrast, urban settings with greater exposure to HIV awareness campaigns and stronger peer support networks may foster higher disclosure rates, as seen in Debre Markos and Jimma.

In the current study, having a prior counseling about HIV testing with sexual partners has been identified as a significant determinant affecting the disclosure of HIV-positive status among PLWHA. Clients who discussed HIV testing with their partners before the test were 7.86 times more likely to disclose their HIV-positive status compared to those who did not receive precounseling. This increased likelihood may be due to the fact that discussing the issue beforehand prepares clients for the potential outcomes, making it easier for them to accept a positive result. Such discussions help couples understand their HIV risks and set realistic expectations, leading to smoother acceptance of test results when disclosed by their partner. This finding aligns with previous studies [7, 26] suggesting that receiving counseling about HIV/AIDS not only encourages disclosure but also enhances awareness and promotes behavioral changes related to disclosure.

This study found that compared to unmarried HIV-positive adults, married individuals had higher odds of disclosure (AOR: 9.32). This higher likelihood might be attributed to the commitment married individuals have to protect their spouse from HIV transmission and the daily interactions inherent in marital relationships, which may encourage more open communication about HIV status. This finding aligns with a study conducted in Holeta, Central Ethiopia [20, 27].

The current study found that HIV-positive adults exposed to initiating factors such as social media (TV, radio) and interactions with healthcare providers were 7.18 times more likely to disclose their HIV-positive status compared to those without such initiating factors. This increase in disclosure likelihood may be due to the heightened awareness and understanding of the importance of disclosure that these initiating factors provide. This finding is consistent with a study conducted in Mekelle, Ethiopia [13].

In the current study, individuals who underwent peer-initiated VCT had higher odds of disclosure compared to those who underwent provider-initiated VCT (AOR: 6.44 (2.43, 17.07). This high odds of disclosure may be due to the influence of peer support and advice, which can positively affect participants' attitudes and perceptions toward disclosure. This finding is consistent with studies conducted in the Amhara region, Mekelle, and Jimma, Ethiopia [4, 13, 28].

In the current study, adults who perceived HIV-related stigma were 79% less likely to disclose their HIV-positive status to their partner compared to those who did not perceive such stigma. This lower likelihood may be due to stigma creating prejudice and misconceptions, labeling individuals as socially unacceptable. Additionally, stigmatized individuals might fear negative consequences of disclosure, such as depression, social withdrawal, psychological stress, and loss of family support. This finding is consistent with the CDC report fact sheet and a study conducted in Jimma, Ethiopia [28–30].

This study also found that individuals who had clinical symptoms during their HIV status test were 22.12 times more likely to disclose their HIV-positive status to their partner compared to those who did not have clinical symptoms. This increased likelihood may be because symptomatic HIV patients often seek more support and are more open about their condition, leading to higher rates of disclosure. The following table presents a comparative analysis of HIV status disclosure rates from our study and prior regional studies. It highlights variations in disclosure percentages and key influencing factors, such as ART adherence, social support, and stigma. The differences in rates are contextualized based on healthcare system strength, psychosocial influences, and programmatic interventions (see Table 6).

## 5. Conclusion

The study found that the rate of HIV serostatus disclosure to sexual partners among HIV-positive adults was 67.9% (95% CI: 63.5%, 72.5%). This implies that HIV status disclosure status is a considerable public health problem among adults living in the study area. Several factors were significantly associated with increased likelihood of disclosure, including pretest counseling, marital status, the presence of initiating factors, type of HIV testing, perception of HIV-related stigma, and having clinical symptoms at the time of testing.

Thus, expanding access to and improving the availability and quality of pretest counseling, creating focused interventions for unmarried individual living with HIV to promote candid dialog and disclosure to their partners, utilize peer support, healthcare providers, and social media to encourage disclosure and increase awareness are essential steps. We put plans in place to lessen stigma through community involvement and education. HIV status disclosure rates can be raised by supporting peer support programs, peer-initiated testing to increase disclosure rates, and making sure that those who arrive with clinical symptoms receive thorough care and counseling. “These findings have important implications for public health interventions and clinical practice. Elucidation of the factors associated with HIV disclosure may help develop culturally sensitive counseling strategies and peer support programs that empower individuals to disclose safely. Healthcare providers should integrate tailored disclosure support into standard HIV care, while policymakers and community-based organizations should collaborate to decrease stigma and promote enabling environments that support disclosure. Future interventions should focus more on the improvement of social support systems and the reduction of barriers to disclosure for better individual well-being and improved public health outcomes.”

### 5.1. Limitation of the Study

• This study utilized a cross-sectional design, which limits the ability to establish causal relationships or assess changes in HIV disclosure patterns over time. Future longitudinal studies are recommended to provide deeper insights into the dynamics of disclosure and associated factors.
• Since stigma perception is inherently subjective, responses may have been influenced by social desirability bias, leading to potential underreporting of stigma-related experiences. Future studies should consider incorporating qualitative methods to explore the nuanced and contextual aspects of stigma more deeply.
• We excluded participants who initiated ART recently.

## Figures and Tables

**Table 1:** Variance inflation factor (VIF) for assessing multicollinearity among independent variables included in the analysis.

S.N	Variables	Variance inflation factor (VIF)
1	Sex	6.90
2	Education level	4.9
3	Marital status	3.88
4	Places of residence	4.22
5	Employment status	4.34
6	Occupation	6.55
7	Knowledge of HIV status	4.78
8	Duration since HIV diagnosis	4.8
9	ART adherence	4.67
10	Perceived stigma	2.77
11	Types of VCT	4.67
12	Initiating factors of HIV test	3.3
13	Having children	4.98
14	Relationship with sexual partner	3.6
15	With whom participants did for HIV test	4.92
16	HIV status of sexual partner	4.45
17	Presence of clinical symptoms	3.42
18	Pretest counseling	3.67
19	Number of sexual partner	3.77

**Table 2:** Sociodemographic and personal-related characteristics of HIV-positive adults at Sheger city, Ethiopia, 2023.

Variable	Category	Frequency	Percentage
Sex	Male	176	44.8
	Female	217	55.2

Age	< 24	53	13.5
	25–34	194	49.4
	35–44	105	26.7
	45–54	32	8.1
	> 55	9	2.3

Religion	Orthodox	267	67.9
	Muslim	73	18.6
	Protestant	46	11.7
	Other^∗^	7	1.8

Marital status	Married	247	62.8
	Divorced	32	8.1
	Widowed	40	10.2
	Unmarried	74	18.8

Educational level	No formal education	95	24.2
	Primary	174	44.3
	Secondary	75	19.1
	Certificate and above	49	12.5

Occupation	Govt employee	21	5.3
	Farmer	72	18.3
	Private/own business	94	23.9
	Daily laborer	29	7.4
	Housewife	122	31.0
	Student	41	10.4
	No job	14	3.6

Having children	Yes	132	33.6
	No	261	66.4

^∗^Wakefata, Catholic.

**Table 3:** Psychosocial and sexual characteristics of HIV-positive positive adults at Sheger city, Ethiopia, 2023.

Variables	Category	Frequency	Percentage
Initiating factors of HIV test	Social media	246	62.6
	Health care providers	204	51.9
	Self-initiated	176	55.2

Type of VCT	Routine VCT	85	21.6
	VCT at ANC	123	31.3
	Provider-initiated VCT	107	27.2
	VCT by peer-counseled	78	19.8

Number of sexual partner	Only one	345	87.8
	Two and above	31	7.9
	Other	17	4.3

HIV status of sexual partner	HIV-positive	217	55.2
	HIV-negative	68	17.3
	Unknown status	108	27.5

Relationship with sexual partner	Smooth	280	71.2
	Not smooth	113	28.8

Perceive HIV stigma	Yes	140	35.6
	No	253	64.4

With whom participants did for HIV test	Alone	298	75.8
	With partner	86	21.9
	Family/relatives	9	2.3

**Table 4:** Behavioral and partner reaction characteristics of HIV-positive adults at Sheger city, Ethiopia, 2023.

Variables	Category	Frequency	Percentage
Drinking alcohol	Yes	203	51.7
	No	190	48.3

Khat chewing	Yes	51	13.0
	No	342	87.0

Cigarette smoking	Yes	34	8.7
	No	359	91.3

Talked about HIV/AIDS with your partner before VCT	Yes	196	49.4
	No	197	50.1

Received pretest counseling related to disclosure	Yes	172	43.8
	No	221	56.2

Having clinical symptoms during disclosure	Yes	163	41.5
	No	230	58.5

Engaged in sexual intercourse before disclosing	Yes	13	3.3
	No	380	96.7

Disclosing been helpful	Yes	245	62.3
	No	148	37.7

Used condoms immediately after diagnosis before disclosure	Yes	259	65.9
	No	134	34.1

Family members react to results	They supported me	176	65.9
	Discriminated against me	91	34.1

Annoyed by people after disclosing	Yes	76	28.5
	No	191	71.5

Worried about own HIV Status following disclosure	Yes	57	21.3
	No	210	78.7

Confused following disclosing	Yes	45	16.9
	No	222	83.1

**Table 5:** Factors associated with HIV disclosure status among adults living in Sheger city, Ethiopia, 2023.

Associated factors	Category	HIV status disclosed		COR 95% CI	AOR 95% CI	*p*-value
		Yes (%)	No (%)			
Pretest counseling	Yes	142 (53.2)	30 (23.8)	3.64 (2.26, 5.85)	7.86 (3.61, 17.08)	0.001^∗^
	No	125 (46.8)	96 (76.2)	1	1	

Marital status	Married	192 (71.9)	55 (43.7)	0.26 (0.15, 0.44)	9.322 (2.62, 33.19)	0.01^∗^
	Divorced	15 (5.6)	17 (13.5)	1.02 (0.44, 2.33)	1.2 (0.40, 3.56)	0.747
	Widowed	25 (9.4)	15 (11.9)	0.54 (0.25, 1.18)	3.67 (1.37, 9.85)	0.07
	Unmarried	35 (13.1)	39 (31.0)	1	1	

Presence of initiating factors	Yes	95 (75.4)	31 (24.6)	12.67 (7.6, 21)	7.18 (3.41, 15.01)	0.031^∗^
	No	52 (19.5)	215 (80.5)	1	1	

Types of VCT	Routine VCT	77 (28.8)	8 (6.3)	1	1	
	VCT at ANC	85 (31.8)	38 (30.2)	4.3 (1.89, 9.79)	0.77 (0.25, 2.34)	0.65
	Provider-initiated testing	50 (18.7)	57 (45.2)	11.8 (5.2, 26.9)	2.5 (0.95, 6.6)	0.064
	Peer-initiated testing	55 (20.6)	23 (18.3)	4 (1.67, 9.66)	6.44 (2.43, 17.07)	0.014^∗^

Perception of Stigma	Yes	104 (39.0)	36 (28.6)	1	1	
	No	163 (61.0)	90 (71.4)	0.63 (0.40, 0.99)	0.21 (0.09, 0.47)	0.001

Presence of clinical symptoms	Yes	155 (58.1)	8 (6.3)	20.4 (9.56, 43.5)	22.12 (8.74, 56.2)	0.001^∗^
	No	112 (41.9)	118 (93.7)	1	1	

Abbreviations: AOR = adjusted odds ratio, CI = confidence interval.

^∗^Statistically significant variables.

**Table 6:** Comparative analysis of HIV disclosure rates and key associated factors across study.

Study	Country/region	Magnitude of disclosure (%)	Key factors	Strength of association (AOR, 95% CI)
Current study	Ethiopia (Sheger city)	67.9%	✓ Prior counseling with a partner ✓ Marital status ✓ Exposure to social media & healthcare providers ✓ Peer-initiated VCT ✓ HIV-related stigma ✓ Presence of clinical symptoms	✓ Counseling before HIV testing ↑ disclosure (AOR: 7.86) ✓ Married individuals ↑ disclosure (AOR: 9.32) ✓ Social media and healthcare exposure ↑ disclosure (AOR: 7.18) ✓ Peer-initiated VCT ↓ disclosure (AOR: 0.44) ✓ HIV-related stigma ↓ disclosure (79% less likely) ✓ Presence of clinical symptoms ↑ disclosure (AOR: 22.12)

Study in Uganda [3]	Uganda	62%	✓ ART adherence and partner support	✓ Increased and partner support of ART adherence ↑ disclosure

Study in Amhara, Ethiopia [3]	Ethiopia (Amhara)	73%	✓ relationship status and social support	✓ Types of the relationship and social support ↑ disclosure

Study in Holeta, Ethiopia [7]	Ethiopia (Holeta)	72.1%	✓ Stigma, fear of rejection, and marital status	✓ Married individuals ↑ disclosure (similar finding)

Study in Mekelle, Ethiopia [14]	Ethiopia (Mekelle)	57.4%	✓ Limited ART access, social barriers, and exposure to media	✓ Social media & healthcare exposure ↑ disclosure (similar finding)

Study in Axum, Ethiopia [19]	Ethiopia (Axum)	52.6%	✓ Healthcare quality and disclosure support	✓ Heathcare quality and disclosure support

Study in Bale, Ethiopia [20]	Ethiopia (Bale)	41.8%	✓ Stigma and ART access	✓ Not reported ↑ HIV disclosure to sexual partner

Study in Ho Municipality, Ghana [4]	Ghana	88.7%	✓ Strong healthcare system and policy support	✓ Strong healthcare policy ↑ HIV disclosure to sexual partner

Study in Debre Markos, Ethiopia [10]	Ethiopia (Debre Markos)	92.6%	✓ ART adherence and partner support	✓ ART adherence and partner support↑ HIV disclosure to sexual partner

Study in Jimma University Hospital [21, 25]	Ethiopia (Jimma)	90.8%	✓ Counseling services, peer support, and HIV-related stigma	✓ Peer-initiated VCT ↓ disclosure (similar finding) HIV-related stigma ↓ disclosure (similar finding)

Study in Ambo Hospital, Ethiopia [22]	Ethiopia (Ambo)	86.2%	✓ Health education and social reinforcement	✓ Presence of health education, social reinforcement ↑ HIV disclosure to sexual partner

## Data Availability

All data supporting the findings of this study are available within the paper and its Supporting Information.

## Nomenclature

AOR: Adjusted odds ratio
ART: Antiretroviral therapy
ARV: Antiretroviral therapy
HIV: Human immunodeficiency virus
PLHIV: People living with human immunodeficiency virus
WHO: World Health Organization

## Supporting Information

Supporting File 1—Questionnaire for HIV disclosure status to partners and its associated factors among HIV-positive adults at Sheger city, Ethiopia, 2023.
