# Supplementary material for: Exploring the HIV Disclosure Patterns to Sexual Partners and Associated Factors Among HIV-Positive Adults in Sheger City, Ethiopia: A Multicenter Study
Source: AIDS Res Treat. 2025 Apr 17;2025:4117734. doi: 10.1155/arat/4117734 (PMC12021478; doi:10.1155/arat/4117734)
Supplement: Supporting Information — Additional supporting information can be found online in the Supporting Information section. [file 4117734.f1.pdf]

Annexes

Annex I. Study information sheet

This sheet is to be read for the participants of the study.

Good morning/afternoon, my name is ____________________and I am one of the data collectors for the study being conducted to "assess HIV disclosure status to partners and its associated factors among HIV positive adults at sheger city, Ethiopia, 2023." You are selected scientifically to be participant of this study if you give me consent after you have understood the following information sheet:

Title of the study: a cross sectional study on HIV disclosure status to partners and its associated factors among HIV positive adults at sheger city, Ethiopia, 2023, Ethiopia.

Back ground of the study: Disclosure of HIV positive status to partners and its associated factors among HIV Positive Adults is important for the prevention and control of HIV/AIDS. Thus, this study examined HIV disclosure status and its associated factors among HIV Positive Adults.

Objective of the study:-HIV disclosure status to partners and its associated factors among HIV positive adults following ART follow up at sheger city, Ethiopia, 2023.

Benefit of the study:-

The participant will not get any direct benefit for being participant

The result can be used as a baseline for further studies that can be done in this Zone.

The result will be used to design prevention and control measures of HIV/AIDS.

The result will be disseminated to the Finfinne Special Zonal Health Office.

Harm of the study: the study has no any harm except that participant will spend up to 20- 25 minutes in the interview.

Rights of the participant: -participation has full right not participate the participant can stop participating in the study at any time the participant can skip question which she/he does not want to respond during the interview, the participant can ask questions which are not clear Confidentiality: - the secrecy of any information forwarded will be maintained.

Annex II. Consent form

I, the selected participant, heard the information in the study information sheet and understood the purpose, benefit, and what is required from me and what happen to me if itake part in the study. I understood that all the information regarding me, like name and all answers given by me must not be transferred to the third party. I also understand that i can decide whether or not to take part in the study or even withdraw from the study at any time.

The participant sign________________________

Name of theiInterviewer _____________Signature _______date _______

Name of the supervisor _______________Signature _______date ______

Address of the investigator:

Mobile = 09 11 11 94 65 / 09 47 27 87 47

Email = hmesfin91@gmail.com

"Thank you for your willingness to participate in this study!"

Annex III:- English Language questionnaire

Questionnaire for HIV disclosure status to partners and its associated factors among HIV positive adults at sheger city, Ethiopia, 2023

name of interviewer:____________________		Name of the Health Facility:________________	
Study Number___________________		Date of interview: _____/_______/______	
S. No	**Questions and Filters**	**Response & coding categories**	Skip
**Section I: Socio- demographic characteristics of the participants**			
101	How old are you?	___________	
102	Sex?	1. Male 2. Female	
103	Residence of Respondents?	1. Male 2. Female	
104	What is your religious denomination?	___________________	
105	What is your ethnic group?	____________	
106	What is your current marital status?	_____________	
107	What was your education Level during Your HIV/AIDS test period Test Period?	___________ _	
108	Do you have a children?	1. Yes 2. No →	Skip to Q. 111
109	If said yes above, how many children do you have?	___________	
110	What is your major paid occupation currently? (Whatever you do to earn money)?	_____________	
111	How much is your household income, including your own?	_________	
**Part II:-Questions regarding psychosocial and sexual characteristics of the respondents**			

S. No	**Question**	**Coding categories**	**Code**	**Skip**
201	What is your Initiating factor to test for HIV?	1. Heard on radio/TV<br>2. Health provider recommendation<br>3. Self-initiated 4. None		
202	Do you have receiving pre-test counselling related to disclosure?	1. Yes 2. No		
203	Have any Clinical symptoms for disclosure?	Yes 2. No		
204	What was the type of VCT service you Had under taken during HIV diagnosis?	1, Routine VCT<br>2, VCT at ANC	3, Provider initiated VCT service<br>4, VCT by peer counselor	
205	With whom did you test for HIV?	1. Alone<br>2. With my partner	3. Other specify____	
206	What was your CD4 count when you begin ART if you know it?		_______________________<br>_	
207	How many sexual partners did you have when you have done HIV test?	1. Only One<br>2. Two	3. I don’t remember<br>4. More than Two	
208	Which substance did you use during your HIV diagnosis period?	1. Alcohol<br>2. Chat	3. Cigarette<br>4. None	
209	What is your Sexual Partner status?	1. HIV Positive<br>2. HIV Negative	3. Unknown HIV serostatus	
210	Duration of Sexual Relation With Sexual Partner?	1. Less tha 1 year	3. 3-4 Years<br>4. 5 years and above	

		2. 2-3 Years		
211	Are you a member of anti-HIV/AIDS association?	1, Yes	2, No	
212	Do you participating in voluntary groups?	1, Yes	2, No	
213	Do you Perceived HIV related stigma?	1, Yes	2, No	
214	What's your relationship with sexual partner?	1. Smooth	2. Not Smooth	
**Part III**:- **Questioners regarding HIV status disclosure among HIV positive ART users**				
301	Have you ever talked about HIV/AIDS related topics with your partner before VCT?	1, Yes 2, No<br>3, I did not remember it		
302	Have you ever talked about VCT with your sexual partner before your HIV test result?	1, Yes 2, No<br>3, I did not remember it		
303	Did you have willing to disclose your HIV status when you have done VCT	1, Yes	2, No	
304	Have you disclosed your HIV serostatus to your sexual partner?	1, Yes	2, No	3.5
305	Why you disclosed you HIV sero-status to your sexual partner	1, To prevent Virus transmission<br>2, To make partner to be examined and know his status<br>3, To prevent virus transmission to new born<br>4, To gain support from partner		
306	If you had disclosed your HIV	1. Immediately after VCT test result		

	serostatus when have you disclosed?	2. After one week of test result<br>3. Within 1 week & 1 month<br>4. Within 1 month- 2 month<br>5. Within 2 months- 7 month<br>6. 7-12 month 7. 1-2 Years<br>7. 2-4 Years 9. 4 Years and Above		
307	For whom have you disclosed your HIV test result except your sexual partner	1, Mother<br>2, Father<br>3, Sister<br>4, Brother	6, Relatives<br>7, Close friends<br>8, Religious members<br>9, PLWHA members<br>10, Others ___________	
308	Do you have an intention to disclose more to people?	1, Yes	2, No	
309	Why you have not disclosed to anybody mentioned above or to the whole individuals who need to be disclosed?	1, Fear of partner violence<br>2, Fear of loss of confidentiality<br>3, Fear of divorce<br>4, Stigma and Discrimination<br>5, Health professionals not informed me to notify my partner<br>6, Loss of support from partner/Family<br>7,fear of accusation for in fidelity		
310	Did you develop depression after disclosure?	1. Yes	2. No	
311	Have you engaged in sexual intercourse before	1. Yes	3, I don't remember	

	disclosing your serostatus to your sexual partner?	2. No		
312	Has disclosing our status been helpful in living with HIV?	1, Yes	2, No	
313	Did you have used condoms immediately following your HIV status diagnosis before disclosure?	1, Yes<br>2, No	3, Not remember	
314	Why you did use condom	1, To prevent pregnancy<br>2, To prevent virus transmission to partner<br>3, Not wanted additional child		
315	Why not used condoms	1, Partner was HIV positive 3, Need of child<br>2, Use of decrease satisfaction 4, Religious prohibition		
**Part IV:- Questions regarding sexual partner reaction following HIV sero-status disclosure**				
401	How did the family members react to your HIV positive results?	1, they supported me 2, discriminated against me<br>3, I don't know 4, Others specify…		
402	Had you got reassurance from the partner, friends, family, neighbors, relatives, working environment and friends?	1. Often got<br>2. Got no reassurance reassurance<br>3. Some got reassurance		
403	Are you annoyed by people after disclosing your HIV sero-status?	1. Yes	2. No	
404	Are you confused following disclosing your HIV sero-status?	1, Yes 2, No	3, Do not remember	

405	When you had disclosed your HIV sero-status did the individual you disclosed your sero-status cried	1, Yes	2, No	
406	Had anybody that had known your sero-status talked about leaving usual relationship?	1, Yes	2, No	
407	Had your sexual partner worried about his/her own HIV serostatus following disclosure of your HIV test result?	1. Yes<br>2. No	3. I don't know	
408	After your HIV serostatus disclosure had anybody left away from you?	1, Yes	2, No	
409	Had your sexual partner after disclosure threatened you?	1, Yes	2, No	
410	Had your partner beaten you following Disclosure of serostatus?	1. Yes as more often<br>2. Beaten as usual<br>3. No he has not beaten till know		

Section V;-Perceived stigma and discrimination

This set of questions asks about some of your feelings and opinions as to how people with HIV feel and how they are treated. There is no right or wrong answer. Please feel free to tell us what you think. Could you tell me if you agree or disagree with the following statements

		Strongly disagre	Disagre	Agree	Strongly agree
501	In many areas of my life, no one knows that I have HIV	1=☐	2=☐	3=☐	4=☐

502	I feel guilty because I have HIV	1=☐	2=☐	3=☐	4=☐
503	People's attitudes about HIV make me feel worse about myself	1=☐	2=☐	3=☐	4=☐
504	Telling someone I have HIV is risky	1=☐	2=☐	3=☐	4=☐
505	People with HIV lose their jobs when their employers find out	1=☐	2=☐	3=☐	4=☐
506	I work hard to keep my HIV a secret	1=☐	2=☐	3=☐	4=☐
507	I feel I am not as good a person as others because I have HIV	1=☐	2=☐	3=☐	4=☐
508	I never feel ashamed of having HIV	4=☐	3=☐	2=☐	1=☐
509	People with HIV are treated like outcasts	1=☐	2=☐	3=☐	4=☐
510	Most people believe that a person who has HIV is dirty	1=☐	2=☐	3=☐	4=☐
511	It is easier to avoid new friendships than worry about telling someone that I have HIV	1=☐	2=☐	3=☐	4=☐
512	Having HIV makes me feel unclean	1=☐	2=☐	3=☐	4=☐
513	Since learning I have HIV, I feel set apart and isolated from the rest of the world	1=☐	2=☐	3=☐	4=☐
514	Most people think that a person with HIV is disgusting	1=☐	2=☐	3=☐	4=☐
515	Having HIV makes me feel that I'm a bad person	1=☐	2=☐	3=☐	4=☐
516	Most people with HIV are rejected when others find out	1=☐	2=☐	3=☐	4=☐
517	I am very careful who I tell that I have HIV	1=☐	2=☐	3=☐	4=☐

518	Some people who know I have HIV have grown more distant	1=☐	2=☐	3=☐	4=☐
519	Since learning I have HIV, I worry about people discriminating against me	1=☐	2=☐	3=☐	4=☐
520	Most people are uncomfortable around someone	1=☐	2=☐	3=☐	4=☐
521	I never feel the need to hide the fact that I have HIV	4=☐	3=☐	2=☐	1=☐
522	I worry that people may judge me when they learn I have HIV	1=☐	2=☐	3=☐	4=☐
523	Having HIV in my body is disgusting to me	1=☐	2=☐	3=☐	4=☐

Section VI. Social Support (OSLO SOCIAL SUPPORT SCALE)

601	How many people are so close to you that you can count on them if you have serious problems?	None	1	
		1 or 2	2	
		3-5	3	
		6 or more	4	
602	How much concern do people show in what you are doing?	A lot of concern and interest	5	
		Some concern and interest	4	
		Uncertain	3	
		Little concern and interest	2	
		No concern and interest	1	
603	How easy can you get practical help from neighbors if you should need it?	Very easy	5	
		Easy	4	
		Possible	3	
		Difficult	2	

		Very difficult	1	

Annex IV. Afan Oromo questionnaire

Yuniiversiitii Ambootti, Kolleejii Saayinsii Fayyaa, Mana barumsaa Fayyaa Hawasaatti qoo‟annoo waa‟ee bu’aa/firii qorannoo dhiigaa dhukkubaa HIV/AIDS ifaa baasu fi Rakkoolee kanaan walqabatan magaalaa shaggar irratti ta‟uuf gaaffiwwan qophaa‟an.

A. Unka odeeffannoo

Nagaa bultanii/oltanii! Maqaan kiyya____________________________jedhama. Ani hojjetaa/ttu __________________________yoo ta‟u qoo‟annoo waa‟ee bu’aa/firii qorannoo dhiigaa dhukubaa HIV/AIDS ifaa baasu fi rakkoolee kanaan walqabatan Yuniiversiitii Ambootiin geggeeffamaa jiruf akka odeeffanno funanuf filatamera. Isin immoo qoo‟annoo kana keessatti akka hirmaattaniif haala saayinsawaa ta‟en filatamtaniittu; gaaffii tokko tokko ani isin gaafadhuuf deebii naaf kennitu jedheen abdii guddaa qaba.Wanta ani isin hubachiisuu barbaaduu deebiin isin nuuf deebistan/kennitan icciitiin Kan eegamu ta’a. Maqaa keessanii nutti himuun hin barbaachisu. Kana malees mirga guutuu qoo‟annaa kana keessatti hirmaachuu fi hirmaachuu dhiisuu, gaaffii isin hin ilaallanne irra darbuu, akkasumas gaaffii fi deebii itti fufuu yoo hin barbaanne ta’e gidduutti dhaabuuf mirga guutuu qabdu. Gaaffiin tokko tokko waa‟ee jireenya dhuunfaa keessanii waan ilaallatuuf deebisuuf ulfaataa ta‟u ni danda‟a haata'u malee galma ga'umsa kaayyoo qorannoo kanaaf muuxannoon keessan baay‟ee barbaachisaa fi kan bu‟aa olaanaa qabudha. Gaaffii fi deebiin Kun tilmaamaan daqiiqaa 20-25 fudhata. Gaaffii gaafattan qabduu?

Baay‟ee galatoomaa!

Qo‟annaa kana keessatti hirmaachuuf fedha qabdaa?

Eeyyee ☐ Lakkii ☐

Eeyyee lakki Deebiin keessan „Eeyyee‟ yoo ta‟e gara gaaffii fi deebii koottan darba.

B. Unka waliigaltee

Ani mallattoo kiyya armaan gaditti kanan kaaye namoonni qoo‟annoo kana geggeessaa jiran faayidaa qoo‟annoo kana ifa naa godhaniiru akkasumas gaaffiin beekuu fi na ilaallatu akkan deebisuuf ifa naa godhaniiru. Mata dureen qoo‟annoo kanaas waa‟ee bu’aa/firii qorannoo dhiigaa dhukkubaa HIV/AIDS ifaa baasu fi rakkolee kanaan walqabatan ta‟u isaa natti himameera. Kana malees odeeffannoon ani kennu qoo‟annaa kana qofaaf akka itti fayyadaman, icciitiin akka ta‟u natti himameera. Gaaffii fi deebii keessatti hirmaachuu fi hrmaachuu dhiisuu akkan danda‟u, gaaffii deebisuu hin barbaadne akkan irra darbuu danda‟us natti himameera. Yeroon barbaadettis gaaffii gidduutti dhiisuuf mirga akkan qabu naaf ibsameera. Odeeffannoo armaan olii irratti hunda‟uudhaan, qoo‟annoo kana keessatti fedhii kiyyaan irratti hirmaachuuf walii galuu koo mallattoo kootiin nan mirkaneessa.

Mallattoo___________________________

Maqaa gaafataa_______________________ Mallattoo __________________________

Maqaa too‟ataa ___________________________Mallattoo____________________________

Guyyaa_______________

Teessoo qorataa:

Bilbila = +2519 11 11 94 65 / +2519 47 27 87 47

Email = hmesfin91@gmail.com

“Hirmaannaa keessaniif daran galatoomaa!

Gaaffii hiikaa afaan oromoon qopha’ee

**Maqaa Ragaa Sassabaa:____________________**		Maqaa Dhaabbata Fayyaa **:________________**	
**Lakk Qo’annaa___________________**		Guyyaa gaaffii fi deebii **: _____/______/____**	
Lk	Gaaffilee	Deebii Hirmaattotaa	Gara_darbi
**Kutaa 1:- Gaaffilee dhimma dinagddee fi hawwaasummaatiin wal qabatan**			
101	Umuriin keessan hangami?	Waggaa _____________	
102	Saala?	1, Dhiira 2, Dubartii	
103	Eessa jiraachaa jirta?	1, Magaala 2, Baadiyyaa	
104	Amantiin kee maali?	_________________	
105	Sabni kee maalidha?	__________________	
106	Haalli gaa’ila yeroo ammaa maal fakkaata?	_________________	
107	Yemmuu qorannoo HIV taasiftu sadarkaan barnoota kee hangam turee?	___________________________	
108	Ijoollee qabdaa?	1, Eeyyee 2, Lakkii →	Darbi Gara 110
109	Eeyyee yoo jedhan Ijoollee meeaqa qabdu?	________________	

110	Maddii galii kee beekaman qarshii itti argattuu maaliidha	____________________	
111	Galii kee kan ji'aa kan maatii dabalatee meeqa argattuu?	__________ (Birr/Ji'aan)	
**Kutaa II:-Gaaffilee waa'ee Xinsammuu hawasummaa, Tajajila Yaala fi Amaloota saalqunnamtii hirmaata waliin walqabatu**			
201	Qorannoon HIV yoom isiniif taasifame (galmeen tajajilama haa ilaalamu)?	_________(Ji'a, Waggaa)	
202	Qorannoo HIV kana akka taasiftu wantii si kakaasee maalii?	1, TV/Raadiyoo irraan dhaga'ee<br>2, Gorsa Ogeessa Fayyarraa<br>3, Kaka'umsa keessaa kootoin	
203	Yeroo qorannoo HIV taasiftu tajajila gosa kamiif deemtati tajajila qorannoo kana argattee?	1, Tajajile Idilee Qorannoo fedhii<br>2, Qorannoo fedhii yeroo hordoffii ulfa marsa da'umsa duraa<br>3, Gorsa ogumma argadheen karaa Qorannoo fedhii<br>4, karaa Qorannoo fedhii gorsa hiriyaa booda	
204	Eenyuu waliin qorannoo HIV taasifte?	1, Kophaa Kooo 2, Hiriyaa koo waliin<br>3, kan biro Ibsii_________	
205	Tajajila Yaala qoricha farraa HIV yoom jalqabdee?	____________________	
206	Yemmuu qoricha yaala HIV eegaltu sanatti baay'inni CD4 kee hangam turee yoo yaadattee	__________________	
207	Yemmuu qorannoo HIV taasiftu sanatti hiriyaa saalqunnamtii meeqa qabda truree	1, Tokko qofa 2, Lama<br>3, Lama ol	
208	Wanoota armaan gadii keessaa kam fayyadamta yemmuu qorannoo HIV taasftu sanatti	1, Alkoolii 2, Caatii<br>3, Sigaaraa 4, Homaa	

209	Yemmuu qorannoo HIV taasift hiriyaa kee waliin hariroo gaarii qabda turtee?	1, Eeyyee 2, Lakkii	
210	Dhiignii hiriyaa kee saalqunnamtii maaliidha	1, HIV Positivii 2, HIV Negativii<br>3, hin beekamu	
211	Ati miseensa waldaa namoota HIV waliin jiraataniiti?	1, Eeyyee 2, Lakkii	
212	Hojiwwaan fedhii garee addaa addaa keessatti ni hirmaatta?	1, Eeyyee 2, Lakkii	
213	Miirrii Loogii namoota biraan isin irraa ga'aa jiraachu sitti dhaga'amee jira?	1, Eeyyee 2, Lakkii	
214	Miirrii namoota biraan addaa baafamu kee sitti dhaga'ama?	1, Eeyyee 2, Lakkii	
215	Hariiroon ati nama biro waliin qabdu maal fakkaata?	1, Hiriyumma/gaarii<br>2, Qoodii/Loogii	
**Kutaa III:- Gaaffilee waa'ee namoonnii HIV dhan qabamanii qorichaa fudhachaa jiran hangam namoota biroof akka of ibsaa jiran gaafatu**			
301	Osoo qorannoo HIV fedhii irraatti hundaa'ee hin taasisin dura waa'ila kee waliin mata duree HIV dhan walqabatu irraatti mari'attee jirta?	1, Eeyyee 2, Lakkii<br>3, hin yaadadhu	
302	Osoo bu'aa qorannoo HIV hin barin dura hiriyaa kee saalqunnamtii waliin waa'ee HIV irraatti mara'attee jirta?	1, Eeyyee 2, Lakkii<br>3, hin yaadadhu	
303	Yemmuu qorannoo HIV taasiftee sanatti HIV qabachuu kee nama biraatti dabarsitee himuuf fedhii qabda turtee?	1, Eeyyee 2, Lakkii	
304	Dhiiga kee keessa HIV'n jiraachuu isaa hiriyaa saalqunnamtii keetif of beeksisftee jirtaa?	1, Eeyyee 2, Lakkii	
305	HIV qabachuu kee maliif	1, Vaayiraasichii akka hin dabaarree ittisuuf	

	hiriyaa saalqunnamtii keetitti himtee?	2, Hiriyaan kee qoratamee akka of baruuf<br>3, Vaayiraasichii gara daa'ima haaraa dhalattuuti akka hin dabaarree ittisuuf<br>4, Hiriyaa kiyyaa irraa deeggarsa argachuuf	
306	HIV qabachuu kee of ibsitee jirta yoo ta'ee yoom of ibsite?	1,osoo hin turiin akkuma bu'aan qorannoo fedhii irraatti hunda'ee ergaa naaf taasifame<br>2, Torbee tokko booddee akkuma bua'an qorannoo natti himamee<br>3, Torbee tokko fi ji'a tokko giddutti<br>4, Ji'a tokko fi ji'a Lama giddutti<br>5, Ji'a Lama fi ji'a Torba giddutti<br>6, Ji'a 7-12 7, Waggaa 1-2<br>8, Waggaa 2-4 9, Wagga 4-9 fi Isaa ol	
307	Hiriyaa saalqunnamtttin alatti eenyuu eenyuf HIV qabachuu kee itti of ibsitee	1, Haadhaa 5, Firoottan koo<br>2, Abbaa koo 6, Hiriyaa natti dhihaatan<br>3, Obbooleetti koo 7, Namoota amanta<br>4, Obboleessaa koo 8, Namoota HIV waliin jiratana	
308	HIV qabachuu kee namoota dabalataf of ibsuuf karoora qabda?	1, Eeyyee 2, Lakkii	
309	HIV qabachuu kee maliif of ibsuu dhiiftee yeroo sana ykn hanga ammaa?	1, Sodaa lola waa'ila koo waliin na qunnamu<br>2, Sodaa iccitiin na jala bahu malaa<br>3, Sodaa hiikkaa gaa'ila<br>4, Sodaa Loogii fi dhiibamu narraa gahu	

		5, Ogeessii fayya akkan waa'ila kooti himuu qabu natti hin himnee<br>6, Sodaa deeggrsa dhabuu maatiirraa<br>7, Sodaa quqlullumma dhabuu	
310	Erga HIV qabachuu kee of ibsitee booda mirrii muka'uu sitti dhaga'ameera?	1, Eeyyee 2, Lakkii	
311	HIV qabachuu kee erga bartee osoo hiriyaa saalqunnamtii keetti hin himin walqunnamtii saalaa rawwattee jirta?	1, Eeyyee 2, Lakkii	
312	HIV walin akka jiraachaa jirtu of saaxiluun bu'aa ni qaba jettee yaaddaa?	1, Eeyyee 2, Lakkii	
313	Osoo HIV hin qoratamiin dura yeroo walqunnamtii saala koondomii fayyadamaa turtee?	1, Eeyyee 2, Lakkii	
314	HIV qoratamtee ergaa HIV qabachuu kee bartee booda osoo HIV qabachuu kee of hin ibsin duraa yeroo saalqunnamtii rawwattuu kondomii fayyadama turtee?	1, Eeyyee 2, Lakkii	
315	Kondoomii maaliif fayyadamta?	1, Ulfa ittisuuf 2, HIV'n akka hiriyaa kootti hin 3 dabarree ittisuuf 4, daa'imnii dabalata akk hin dhalanneef	
316	Koondomii maliif hin fayyadamnee?	3, itti quufinsa walqunnamti saala xiqqeessaa<br>4, Amantaan ni dhorka	
**Kutaa IV:- Gaaffilee Waa'ee HIV'n dhiiga kee keessaa jiraachu ergaa ibsiteefi booda hiriyaa saalqunnamtii/Maatii keessaan irraa deebii akkamii argatan jedhu agarsisuu**			
401	Miseensii maatii kee bu'aa qorannoo HIV positivii kee	1, nan deeggaran	

	akkamiin fudhatee?	2, ofirraa nan dhiiban<br>3,beeku hin dandeenye	
402	Hiriyaa saalqunnamtii, miseensa maatii, firoottan dhihoo, namoota waliin hojjeettuu fi kkf irraa jajjabinnii siif taasifameera	1, naaf taasifameera<br>2, darbee adrbee naf taasifameera<br>3, gonkuma hin argannee	
403	Erga HIV qabachuu kee namootaf o ibsitee booda miirrii aarii addaa addaa sirraa gaheera?	1, Eeyyee 2, Lakkii	
404	HIV qabachuu kee of ibssuu keetin wanti si rifachisee jira?	1, Eeyyee 2, Lakkii<br>3, hin yaadadhu	
405	Yemmuu HIV qabachuu kee waa'ila keetif of ibsitu sanatti namnii tiitiof ibsite ni aaree (Aartee)	1, Eeyyee 2, Lakkii	
406	Namonnii HIV qabachuu kee beekan keessaa hariroo keenya addaa kunna kan jedhan jiru	1, Eeyyee 2, Lakkii	
407	Hiriyaan kee kan saalqunnamtii waa'ee bu'aa dhiigaa ishee/isaa yaadda'eera erga ati HIV qabachuu kee itti himtee booda?	1, Eeyyee 2, Lakkii	
408	Erga HIV qabachuu kee namootatti himtee booda namnii si dhiisee deeme jira	1, Eeyyee 2, Lakkii	
409	Hiriyaan kee saalqunnamtii erga HIV qabachuu kee itti himte booda si sodaachiseera / Sodachistee jirtii?	1, Eeyyee 2, Lakkii	
410	Waa'illii kee erga HIV qabachuu ke yemmu itti himtu si rukutee/rukkuttee yoo ta'ee	1, irree deddebi'ee n rukuteera<br>2, akkuma kana dura na rukuteera<br>3, Lakkiii hanga ammaa nan rukunnee	

411	HIV qabachuu kee yemmuu itti himtu waa'illii kee sitti iyyee jira	1, Eeyyee 2, Lakkii	

Kutaa Shan:-Tilmaamame looga fi Addaa baasuun ilaalu:

Kutaa kana keessaatti gaaffileen galan namoota HIV/AIDS'n dhiiga isaanii keessaa jiru miiraa isaanii fi gargarsaa isaaniif taasifamu ilaachisuudhan miiraa fi yaada isin qaban gaafachuuf gaafannoo qopha'eedha. Deebiin sirrii ta'ee ykn sirrii kan hin taanee hin jiru. Waan yaadan dubbachuudhaf homtu isin hin sodachisin gaaffilee Kanaan gaditti dhihaatan ittin waliigalaa ykn itti walii hin galuu jechuudhan deebisa

		Sirritti itti walii hin galu	Itti walii hin galu	Ittin walii gala	Sirrittan itti waliigal
501	Namoonnii baay'een naannoo jireenya koo jiraatan kamiyyuu HIV'n dhiigaa koo keessaa jiraachu hin beekan	1	2	3	4
502	HIV'n dhiiga koo keessatti argamuun miiraa badii rawwadheetu natti dhaga'ama	1	2	3	4
503	Ilaalchii namonnii HIV irraatti qaban irraa kan ka'ee waa'ee koo yaada badaa akka qabaadhu na taasisee	1	2	3	4
504	HIV'n dhiiga koo keessaa akka jiru namatti himuun balaa qaba	1	2	3	4
505	Hojjeettoonnii HIV'n dhiiga isaanii keessa akka jiru hojjeechistonnii irraatti beeknan hojii irraa ari'amu	1	2	3	4
506	HIV'n dhiiga koo keessaa akka jiru dhoksuuf baay'een of eega	1	2	3	4
507	HIV'n dhiiga koo keessaa waan jiruuf namoota kaanii gadi ta'ee natti fakkaata	1	2	3	4

508	HIV’n dhiiga koo keessaa jiraachuu isaatin itti hin qaana’u	1	2	3	4
509	Namoonni HIV’n dhiiga isaanii keessaa jiru ni ajjeefamu	1	2	3	4
510	Namoonni HIV’n dhiiga isaanii keessaa jiru akka waan namoota baay’ee xureessanitti lakka’amu	1	2	3	4
511	HIV’n dhiiga koo keessaa akka jiru himuu caalaa hariroo koo addaan kutuutu natti salphata	1	2	3	4
512	HIV’n dhiiga koo keessaa jiraachuun qulqullummaan akka natti hin dhaga’amnee naa taasisa	1	2	3	4
513	HIV’n dhiiga koo keessaa jiraachuu isaa yoo na beekan Aduunya kanarraa akkan adda cite fi kophaatti akkan waan hafeetu natti dhaga’ama	1	2	3	4
514	Namoonnii baay’een namoota HIV’n dhiiga isaanii keessaa jiru akka isaan nammota jibbisisoodha jedhanii yaadu	1	2	3	4
515	HIV’n dhiiga koo keessaa jiraachuun ani nama badaa akkan ta’eetti akkan yaadu na taasisa	1	2	3	4
516	HIV’n dhiiga isaanii keessaa jiru irraatti yoo beekame ni jibbamu	1	2	3	4
517	HIV’n dhiiga koo keessaa akka jiru eenyuttu himuuf baay’een of eega	1	2	3	4
518	Namoonnii HIV’n dhiiga koo keessaa jiraachu baran narraa fagaatu	1	2	3	4
519	HIV’n dhiiga koo keessaa jiraachuu caala waa’ee namoota ofirraa addaa na baasantu na dhiphisaa	1	2	3	4

520	Namoonnii baay'een namoota akkasi waliin ta'uun itti hin tolu	1	2	3	4
521	HIV'n dhiiga koo keessaa akka jiru dhoksuun akka barbaachisuu yaadee hin beeku	1	2	3	4
522	HIV/ AIDS akkan qabu namoonnii yoo narratti beekan natti murteessuu jedheen dhiphadha	1	2	3	4
523	Dhiiga koo keessaa HIV /AIDS jiraachuun of na jibbisisa	1	2	3	4

Kutaa Jaha. Deeggarsa hawaasaa (Iskeelii deeggarsa hawaasaa Oslo)

601	Osoo rakkoon dhuunfa sirraa gahee namoota hangamitu si cinaa dhaabbata?	Homtu hin jiru	1	
		1-2	2	
		3-5	3	
		6 fi isaa ol	4	
602	Waan ati gootuu namonin hangam fedhii itti qabu ykn xiyyeeffannoo itti kennuu	Fedhii fi xiyyeeffannoo baay'ee	5	
		Darbee darbee fedhii fi xiyyeeffannoo	4	
		Kan hin beekne	3	
		Fedhii fi xiyyeeffannoo xiqqoo	2	
		Fedhii fi xiyyeeffannoo homtu hin jiru	1	
603	Ollaa kee irraa deeggarsii yemmuu si barbaachisuu haallii deeggarsa itti argachuu ollaa kee akkami?	Baay'ee salphaatti argadha	5	
		Salphaadha	4	
		Ni danda'ama	3	
		Ulfaatadha	2	
		Baay'ee ulfaatadha	1	
